# Quantitative analysis of the core 2D arrangement and distribution of enamel rods in cross‐sections of mandibular mouse incisors

**DOI:** 10.1111/joa.12912

**Published:** 2018-11-13

**Authors:** Charles E. Smith, Yuanyuan Hu, Jan C‐C. Hu, James P. Simmer

**Affiliations:** ^1^ Department of Biologic and Materials Sciences University of Michigan School of Dentistry Ann Arbor MI USA; ^2^ Department of Anatomy & Cell Biology Faculty of Medicine McGill University Montreal QC Canada

**Keywords:** enamel formation, enamel rods, quantification, rod decussation, spatial distribution

## Abstract

Considerable descriptive information about the overall organization of mouse mandibular incisor enamel is available but almost nothing is known about the quantitative characteristics of enamel rod arrangement and distribution in these teeth. This has important implications concerning cell movement during the secretory stage because each ameloblast makes one enamel rod. Knowing how many enamel rods are cut open in a cross‐section of the enamel layer could provide insights into understanding the dynamics of how groups of ameloblasts form the enamel layer. In this study, cross‐sections of fully mineralized enamel were cut on 24 mandibular mouse incisors, polished and etched, and imaged by scanning electron microscopy in backscatter mode. Montaged maps of the entire enamel layer were made at high magnification and the enamel rod profiles in each map were color‐coded based upon rod category. Quantitative analyses of each color layer in the maps were then performed using standard routines available in imagej. The data indicated that that there were on average 7233 ± 575 enamel rod profiles per cross‐section in mandibular incisors of 7‐week‐old mice, with 70% located in the inner enamel layer, 27% located in the outer enamel layer, and 3% positioned near the mesial and lateral cementoenamel junctions. All enamel rod profiles showed progressive increases in tilt angles, some very large in magnitude, from the lateral to mesial sides of the enamel layer, whereas only minor variations in tilt angle were found relative to enamel thickness at given locations across the enamel layer. The decussation angle between alternating rows of rod profiles within the inner enamel layer was fairly constant from the lateral to central labial sides of the enamel layer, but it increased dramatically in the mesial region of the enamel layer. The packing density of all rod profiles decreased from lateral to central labial regions of the enamel layer and then in progressing mesially, decreased slightly (inner enamel, mesial tilt), increased slightly (outer enamel layer) or almost doubled in magnitude (inner enamel, lateral tilt). It was concluded that these variations in rod tilt angle and packing densities are adaptations that allow the tooth to maintain a sharp incisal edge and shovel‐shape as renewing segments formed by around 7200 ameloblasts are brought onto the occluding surface of the tooth by continuous renewal.

## Introduction

For over half a century, the continuously erupting incisors of rats and mice have served as a very useful model system for characterizing major cellular, structural, functional, and chemical events that are crucial to forming fully mineralized dentin and enamel layers (Schour & Massler, 1949; Smith & Nanci, 1989; Smith, 1998; Jernvall & Thesleff, 2012; Kuang‐Hsien Hu et al. 2014; Peterkova et al. 2014; Pugach & Gibson, 2014; Renvoisé & Michon, 2014; Klein et al. 2017; Seidel et al. 2017). It is evident from such literature that several developmental modifications evolved to tooth shape and structural organization of hard tissues to accommodate the sideways as opposed to vertical eruptive tooth movement more typical of most mammalian teeth (Jernvall & Thesleff, 2012; Kuang‐Hsien Hu et al. 2014; Peterkova et al. 2014; Renvoisé & Michon, 2014). Among the modifications made to enamel development were changes in the way ameloblasts organize themselves spatially as they differentiate so they eventually go on to form linear sheets of enamel rods stacked progressively one behind each other, all welded together by interrod enamel matrix (Warshawsky, 1971; Jodaikin et al. 1984; Von Koenigswald, 1985; Boyde, 1989; Martin, 1999; Cox, 2013; Nishikawa, 2017). Each sheet is angled in alternating mesial and lateral directions (decussating) and in the case of current living mice and rats tilted forward within the eruptive plane (angled toward incisal tip of tooth; Warshawsky & Smith, 1971; Von Koenigswald, 1985; Moinichen et al. 1996; Martin, 1999; Cox, 2013; Kuang‐Hsien Hu et al. 2014). This is opposed to the more common situation as seen for example in mouse molars where groups of ameloblast form widely divergent enamel rod patterns at different sites across the crown surface, some of which show decussating arrangements (Boyde, 1989; Lyngstadaas et al. 1998; Von Koenigswald, 2004). This subtle bioengineering modification of forming incisally tilted sheets of enamel rods having alternated side‐to‐side angulations (laminated) achieves two clear purposes: (1) it provides a partial fracture plane along the outer enamel portions of the enamel rods that keeps the incisal tip edges sharp for gnawing and (2) it provides considerable abrasion and especially fracture resistance across the sheeted inner enamel portions and radially oriented outer enamel portions as the enamel layer is worn away by attrition at the incisal edges (Warshawsky & Smith, 1971; Von Koenigswald, 1985; Martin, 1999; Vieytes et al. 2007; Habelitz, 2015; Yilmaz et al. 2015).

Each enamel rod traces the path followed by a single ameloblast (Boyde, 1967, 1969; Smith & Warshawsky, 1977; Risnes et al. 2002; Skobe, 2006). As noted above, a great deal of descriptive information currently exists about ameloblasts and how they form and help mineralize the enamel layer, and how the enamel rods they create form a variety of structural arrangements in two‐ and three‐dimensional space. What has been missing from this literature is any kind of perspective about the quantities of enamel rods cut open in a typical cross‐section from either mouse or rat incisors. Such information would be very useful to advance the understanding the cell population dynamics of amelogenesis, that is, how groups of ameloblasts, rather than single ameloblasts, make the enamel layer (Smith & Warshawsky, 1976, 1977; Cox, 2013). The purpose of this project was to answer one simple question about rat or mouse incisor amelogenesis: Can all rod profiles exposed in a single cross‐sectional (transverse) slice of mature rodent incisor enamel be identified and counted without having a huge variation that would render the results unreliable? We opted to use the continuously erupting mandibular incisors of mice to probe this question, in part because the overall thickness of the enamel layer in young adult mice (7 weeks old) is similar to the thickness of the enamel layer present in juvenile rats (100 g bodyweight), for which a considerable amount of quantitative data about cell renewal is available (Smith & Warshawsky, 1975, 1977), but the diameter and length of the incisors in mice is about one‐half the dimensions in rats, which makes them less tedious to quantify (Moinichen et al. 1996). As this report will document, the answer proved very surprising. Rod profiles in cross‐sections of mandibular mouse incisor enamel cannot only be counted reliably but with an unexpectedly low coefficient of variation across many different incisors.

## Material and methods

### Ethical compliance

All procedures involving animals were reviewed and approved by the IACUC committee at the University of Michigan (UCUCA).

#### Sample preparation

Eighteen 7‐week‐old C57BL/6 wild type mice were anesthetized with isoflurane and perfused for 20 min at room temperature with 4% paraformaldehyde in phosphate‐buffered saline (PBS; 135 mm NaCl, 2.7 mm KCl, 4.3 mm Na_2_HPO_4_, 1.4 mm Na_2_H_2_–PO_4_; pH 7.3). Hemi‐mandibles were dissected from the head and cleaned of muscle and soft tissues, and the bone covering the labial side of the incisors was chipped away using dental tools. The hemi‐mandibles were placed in small glass screw‐top vials containing fresh fixative and rotated overnight at 4 °C. The hemi‐mandibles were washed for another day at 4 °C on the rotator in several changes of PBS (pH 7.3), then dehydrated at room temperature in a graded series of acetone, infiltrated for 5 days in diluted then pure Epon 812 substitute, embedded in rectangular silicone molds, and cured for 48 h at 60 °C. Polymerized Epon blocks containing the embedded hemi‐mandibles were trimmed with a coarse rotary diamond wheel on a Model 650 Low‐Speed Diamond Wheel Saw (South Bay Technology, San Clemente, CA, USA). Small 1‐mm‐wide transverse (cross‐sectional) segments of each mandibular incisor were then cut out with a fine diamond blade (0.15 mm) at a site located 8 mm from the apical end, along a plane that was perpendicular (normal) to the curving labial surface (Level 8 section face, illustrated in Fig. 2 of Hu et al. 2011). A group of 1‐mm‐wide segments were placed with the incisal aspect of the enamel layer face down in 25‐mm‐diameter SeriForm mounting cups (Struers, Ballerup, Denmark), and castolite AC plastic (Woodstock, IL, USA) was added and polymerized overnight at room temperature. The SeriForm blocks were polished with successively finer grades (400, 600, and 800) of silicone carbide paper (South Bay Technology) followed by 16 h of polishing with 1.0‐μm alumina abrasive on a Syntron vibrating polisher. The polished block surfaces were cleaned by sonication, the enamel surfaces etched and rapidly washed in a liberal amount of distilled water three times for 15 s each with 0.1% nitric acid, and air‐dried. The final surfaces were coated with a thin layer of carbon.

#### Backscatter electron imaging and construction of enamel layer photo montages

**Figure 1:**
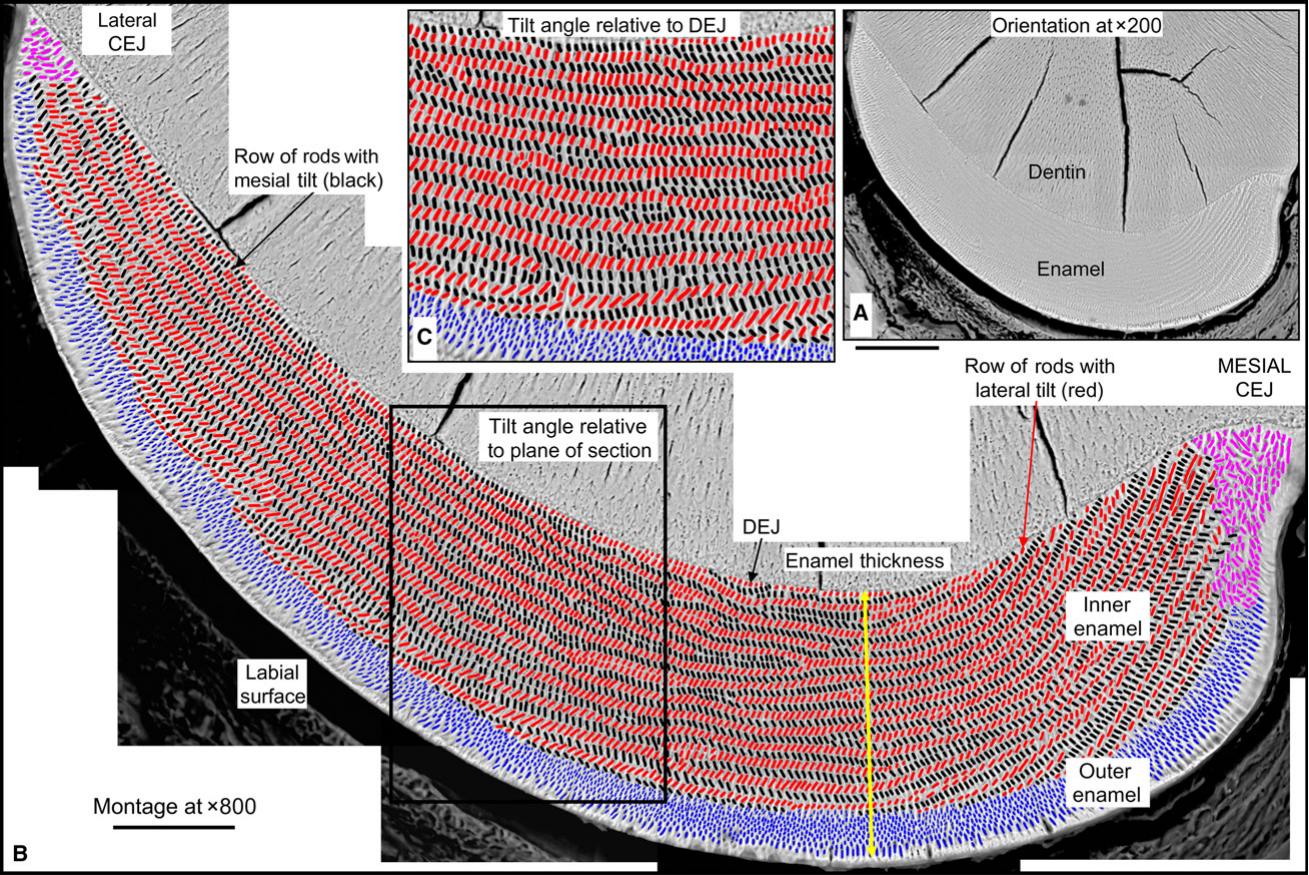
Orientation to the enamel layer and method employed for quantifying enamel rods in cross‐section. Illustration of approach employed for making high magnification maps of the labial side of mandibular mouse incisors from montaged BEI images and for color‐coding enamel rod profiles by their regional distribution within the enamel layer: inner enamel layer with rod profiles having a mesial (black) or lateral (red) tilt, outer enamel layer with rod profiles appearing diamond‐shaped (blue), and irregular rod profiles located near the lateral and mesial cementoenamel junctions (CEJ) (magenta). (A) Low magnification BEI image (×200) of labial side of the mandibular mouse incisor showing location of enamel and dentin in a typical cross‐section of the tooth. The cracks in the dentin are an artifact caused by air drying the tissue slice. (B) A high‐resolution map of the same tooth section shown in (A) made from BEI images photographed at ×800 and montaged together to recreate the whole enamel layer. The site for measuring enamel thickness and regional subdivisions of the enamel layer are indicated. (C) Some quantitative measurements of rod profile tilt angles were made by cropping out areas so their edges were oriented parallel to the DEJ rather than within the plane of the cross‐section (box in B). Scale bars: (A) 100 μm, (B) 50 μm.

The enamel layer covering the labial side of each incisor segment was identified and photographed at low magnification (×200) as a single image at 25 kV using a Hitachi S‐3000N variable pressure scanning electron microscope in the backscatter mode (BEI; Fig. 1A). Then, starting in the region of the mesial cementoenamel junction (CEJ), a series of overlapping high magnification (×800) images were taken across the entire face of the enamel layer from the lateral to the mesial CEJ (Fig. 1B). This process was repeated for enamel samples prepared from the right mandibular incisors of 18 mice. In addition, the enamel layer covering the left mandibular incisors from six mice were also photographed. Each group of 9–12 overlapping high magnification images of the enamel layer on each incisor was placed into separate layers in a single large photoshop file and aligned to recreate a large continuous image of the entire enamel layer on each incisor (Fig. 1B). The completed montages were assessed for accuracy of high magnification alignments by comparing them against the single low magnification images initially taken of the enamel layer on each incisor. A final merged image was created, cropped to touch the surface of the enamel layer just lingual to the lateral CEJ and enamel surface at the point of maximum convexity along the labial surface, and either the dentin or enamel surface at the mesial side (which ever projected the most), then saved in TIF format for each montage.

#### Color‐coding of enamel rods and quantitative analyses using ImageJ

A four‐color coding scheme was used to assign enamel rods into various groupings depending upon their regional location within the enamel layer. These were BLACK for oval rod profiles within the inner enamel layer having a tilt pointed toward the mesial cementoenamel junction, RED for oval rod profiles within the inner enamel layer having a tilt pointed toward the lateral cementoenamel junction, BLUE for diamond‐shaped rod profiles forming the outer enamel layer, and MAGENTA for disorganized rod profiles located near the mesial and lateral CEJ (Moinichen et al. 1996; Fig. 1B,C). To do this, the TIF file that contained the montaged image of the entire enamel layer on each incisor was brought into photoshop and four new imaging layers were created, one for each color. Working at about ×300 enlargement, the sectioned profiles of the enamel rods were outlined and filled with the appropriate color for each category on its appropriate image layer. When completed, either an all‐color image overlaid on the original montage could be saved by layer merging for illustration purposes or a color map for each enamel rod grouping could be saved individually for quantitative analyses in imagej (https://imagej.nih.gov/ij/). Single color maps representing the locations of enamel rod profiles in the inner enamel layer having either a mesial or a lateral tilt were brought into imagej, converted to an 8‐bit grayscale image, and a threshold was defined which best matched the outline of every rod profile in the color map (in this case black or red). Starting near the central aspect of the enamel layer, a single row of enamel rod profiles was identified and outlined with an irregular polygon from its lateral to mesial sides. The ‘Analyze Particles…’ function with ‘Show Outlines’ was used to compute the profile area, centroid, long and short Feret diameters, and Feret angle for each rod profile contained within the polygonal outline. The results were copied to Microsoft excel and sorted by *x*‐axis coordinate position of the centroid, and the results checked for correct sequencing of rod profiles from lateral to mesial endpoints by comparison with the outlines created by imagej following each particle count. Data were coded by mouse ID (1–18), incisor ID (right or left), tooth ID (1–24), row tilt (mesial or lateral; 1 or 2), row ID (1–max), and rod profile ID (1–max). From these raw data, the distance between rod profiles forming the row (distance between *x*‐ and *y*‐coordinates of centroids in sequence across the row) was computed using the standard formula in coordinate geometry: distance = SQRT[(*X*_2_ − *X*_1_)^2^+(*Y*_2_ − *Y*_1_)^2^]. A second Microsoft excel file was created for each incisor containing summary data for each row of rod profiles forming the inner enamel layer in this tooth. This file included mouse, incisor, tooth, row, and tilt identifiers as well as summary variables defining number of rods per row (RPR), the coordinate locations of the lateral endpoint, midpoint and mesial endpoint and their linear distances from the dentoenamel junction (DEJ) relative to a line drawn perpendicular to the DEJ, length of row as sum of inter‐coordinate centroid distances, and average profile area, average long and short Feret diameters, and average Feret angle across all rod profiles forming each row. The angle function in imagej computes angles relative to the horizontal image plane, and in a counterclockwise direction with 0° in a 3 o'clock position (to the mesial side in the case of this study) and 90° in the noon position. All raw data were collected in pixel units and converted afterwards to μm as required using appropriate ‘pixels per micrometer’ scaling factors (pixels/pixels per μm). The *x*‐ and *y*‐coordinate locations of the centroids of rod profiles in each map were also converted from ‘imaging (real world) coordinates’ to ‘normalized (virtual) coordinates’ using a min/max function to identify the boundaries of a rectangle that best fit the outer boundaries of the enamel layer in each coordinate map. These min/max *x*‐ and *y*‐coordinates could then be used to express rod profile locations as a value ranging from a minimum of 0.0 to a maximum of 1.0 in both *x* and *y* directions. The color maps constructed for rod profiles located in the outer enamel layer and near the CEJs were processed in a similar fashion except that it was impossible to assign rod profiles in these regions to any row arrangement. A nearest neighbor plugin for imagej therefore was used to determine inter‐centroid distances between rod profiles in these regions (https://icme.hpc.msstate.edu/mediawiki/index.php/Nearest_Neighbor_Distances_Calculation_with_imagej). All other variables and coding schemes were the same as described above. Data were loaded from excel files into Version 13 of statistica for windows for graphing and statistical analyses (https://www.tibco.com/products/tibco-statistica). Angle data were analyzed and graphed using Version 12 of ncss for Windows (https://www.ncss.com/software/ncss/). A total of 173 598 enamel rod profiles from 24 incisors of 18 mice were analyzed in this study.

## Results

### Features of gross enamel organization in cross (transverse) sections of mouse incisors

The two‐dimensional (2D) organization of rod and interrod enamel in rat and mouse incisor enamel has been described in detail by several researchers (Warshawsky, 1971; Risnes, 1979; Moinichen et al. 1996) and this classic organization is clearly discernible in medium resolution backscatter scanning electron microscopic images (BEI; Fig. 1). Briefly, mouse and rat incisor enamel consists of two thin and two thicker layers stacked on top of one another from the DEJ to the outer surface. The innermost initial layer contains only a thin coat of inter‐rod‐type enamel which ameloblasts create at the start of amelogenesis (not visible in Fig. 1) and an inner enamel layer contains a long portion of rods angled incisally and arranged in several sequential alternating sheets (rows) of rods traveling from near the DEJ outward in either a mesial or a lateral direction toward the surface along with associated inter‐rod enamel. An outer enamel layer contains a short portion of the rods all angled in an incisal direction sloping towards the enamel surface with associated interrod enamel, and the final enamel layer is also composed of only a thin coat of interrod‐type enamel which ameloblasts produce to terminate the appositional growth phase of amelogenesis (somewhat visible in Fig. 1). Quantitative analyses of cross‐sections from 24 mandibular mouse incisors indicated that, on average, enamel layers were 121 ± 2.7 μm thick at the point of maximum convexity along the central labial side (Table 1). A cross‐section of the enamel layer, on average, contained 7233 ± 575 identifiable rod profiles, of which 70% (~ 5000) were associated with the inner enamel layer (Fig. 1, black and red ovals), 27% (~ 2000) with the outer enamel layer (Fig. 1, blue ovals), and 3% (~ 200) with regions abutting the CEJ (Fig. 1, magenta ovals; summarized in Table 1). The 5096 ± 395 rod profiles forming the inner enamel layer were gathered into 124 ± 15 rows split equally by tilt angle (Table 1). Unexpectedly, a slight but significantly higher number of rod profiles having a mesial tilt were counted compared with rod profiles having a lateral tilt (2687 ± 232, *n* = 64 480 vs. 2409 ± 204, *n* = 57 815; Table 1).

**Table 1:** Final summary data for mandibular incisor cross‐sections cut at Level 8^a^ (grand means ± SD)

1. Enamel thickness	121 ± 2.7 μm
Inner enamel layer	100.0 ± 2.6 μm (83%)
Outer enamel layer	21.0 ± 2.1 μm (17%)
2. Total number of rod profiles in cross‐section	7233 ± 575
Inner enamel layer	5096 ± 395 (70%)
Number with mesial tilt	2687 ± 232
Number with lateral tilt	2409 ± 204^b^
Outer enamel layer	1922 ± 191 (27%)
Sites near CEJs	216 ± 56 (3%)
3. Per cross‐section:	
Total number of rows (inner enamel)	124 ± 15
Number with mesial tilt	62 ± 7
Number with lateral tilt	62 ± 9

a Near gingival margin at labial aspect of incisor; *n* = 24 incisors from 18 mice.

b *P* < 0.0001.

### Rod angulations relative to plane of section across the enamel layer

**Figure 2:**
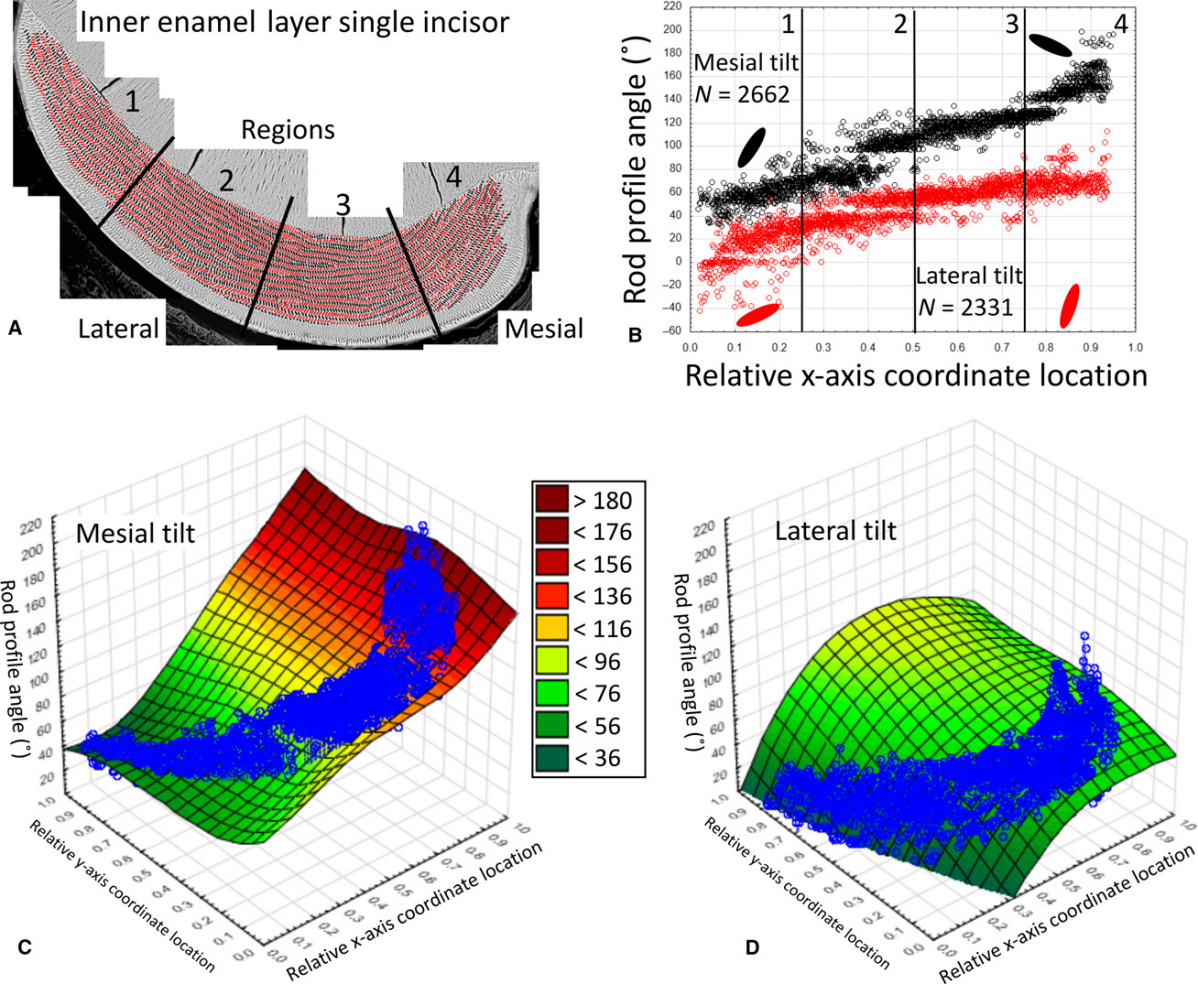
Example data for rod profile angles in the inner enamel layer measured in a single mandibular mouse incisor. Angle data from a single mandibular mouse incisor for rod profiles in the inner enamel layer having a mesial tilt (black) or a lateral tilt (red). (A) Color map for rod profiles. The enamel layer is partitioned into four equally spaced regions from lateral (1) to mesial (4) sides. (B) Graph of rod profile tilt angle (*y*‐axis) vs. location across the face of the cross‐section expressed as virtual coordinates (*x*‐axis). The number of rod profiles plotted from (A) are indicated for each tilt (*N* =) as are schematic representations of the mean rod profile tilts by the large ovals plotted for the lateral (1) and mesial (4) regions. Rod profiles having a mesial tilt (black) show a linear increase in angulations from lateral to mesial sides, whereas rod profiles having a lateral tilt (red) increase initially from lateral to mid lateral regions (1 to 2) and only gradually thereafter (3 and 4). (C,D) Distance weighted least‐squared 3D surface plots of rod profile angulations (*z*‐axis) relative to regional location (*x*‐axis) and location within the thickness of the enamel layer (*y*‐axis) and row tilt (mesial tilt, C; lateral tilt, D). Data values are overlaid to assist visualizing tooth profile outline relative to the more linear surface plot. Some small variations in rod profile angle occur across the thickness of the enamel layer (uniformity of color across the *y*‐axis for a given *x*‐axis coordinate location), but the greatest change in rod profile angle occurs relative to regional location across the face of the cross‐section (change in color relative to *x*‐axis).

**Figure 3:**
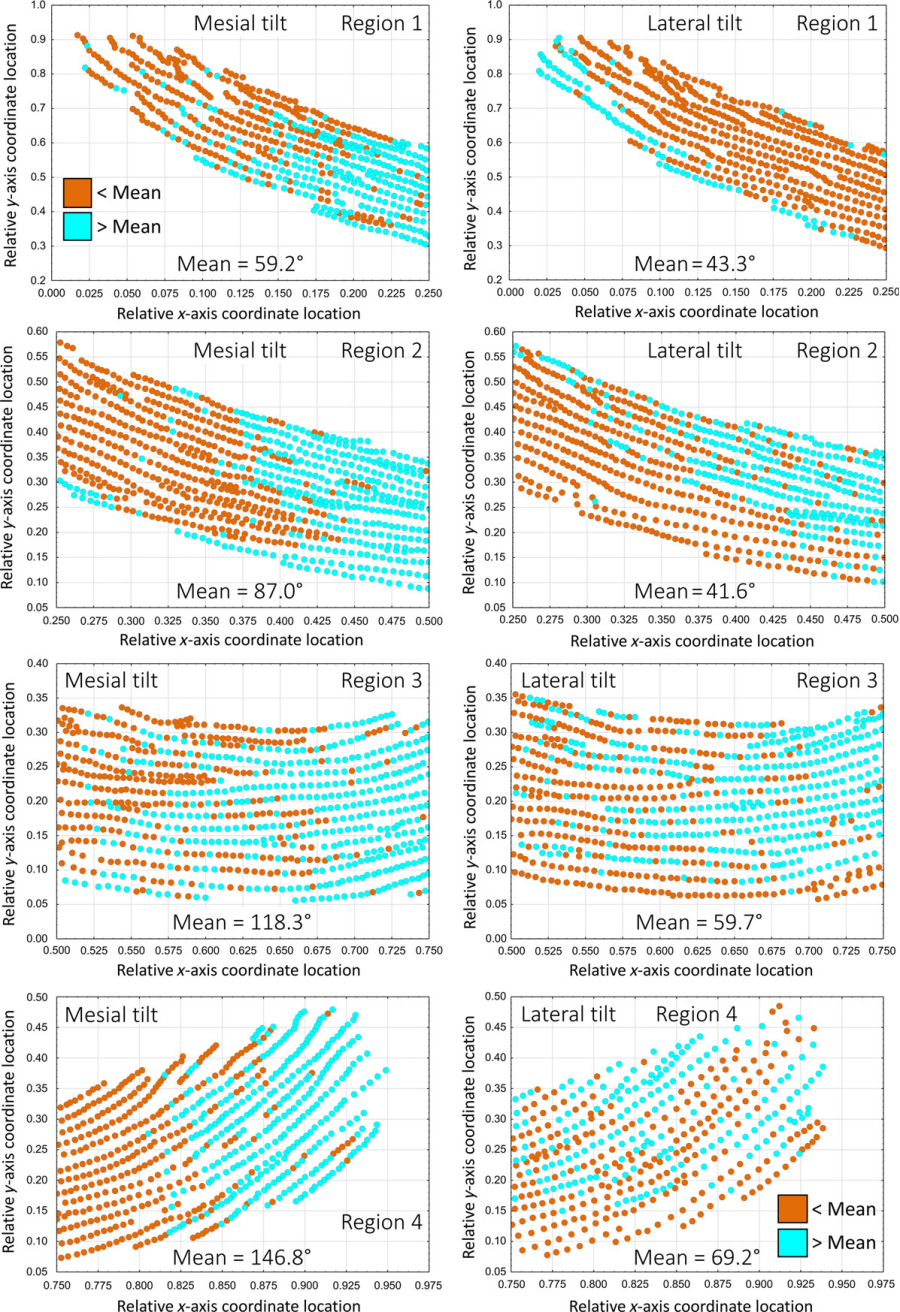
Scatterplots of tilt angle of rod profiles across rows and depth of the inner enamel layer in the lateral (1), mid lateral (2), central labial (3), and mesial (4) regions of the inner enamel layer on a single incisor. Graphs from a single mouse mandibular incisor showing the distribution of rod profile angles for rows having a mesial tilt (left side) or lateral tilt (right side) relative to the mean circular angle (less than, brown; greater than, cyan) computed on a regional basis (lateral, region 1; mid lateral, region 2, central labial, region 3; mesial, region 4). Rod profile angles across different rows or across the thickness of the enamel layer are very variable and show no clear pattern, and less so for rows having a lateral tilt compared with those having a mesial tilt, where changes in the mean circular angle occur more dramatically between regions. The central labial region (3) is the only part of the enamel layer showing some similarities in the distributions of rod profile angles relative to the mean for the two rod tilt categories. In the mesial region (4), rod profiles having a lateral tilt are more widely spaced apart from one another compared with the other regions.

**Figure 4:**
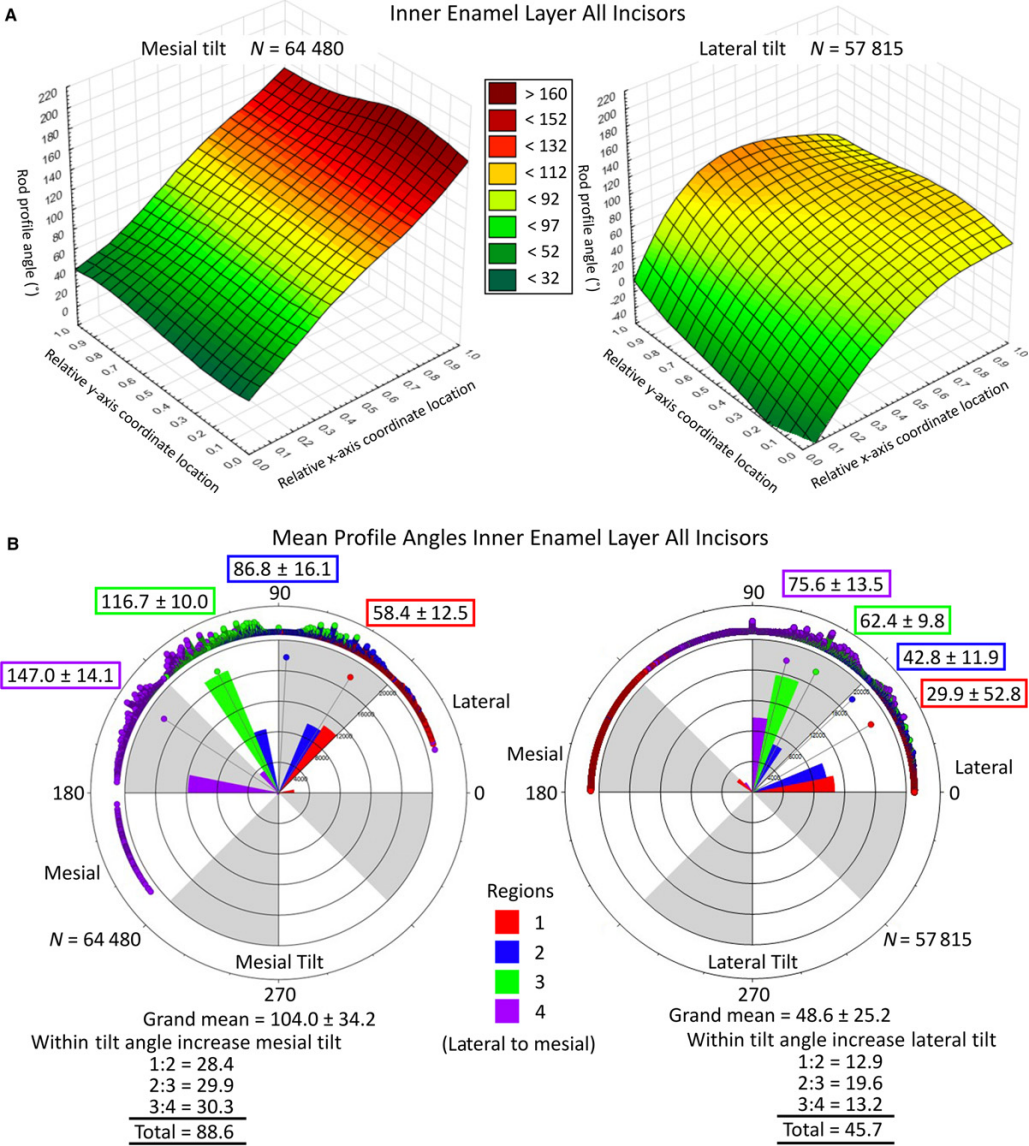
The 3D surface and circular plots of rod profile angles using pooled data from all incisors. The 3D surface plots of rod profile angles across the width and thickness of the inner enamel layer (A). Distance weighted least‐squared 3D surface plots of rod profile angles across the entire inner enamel layer pooled from all mandibular mouse incisors examined in this study (*z*‐axis) plotted relative to regional location (*x*‐axis) and location within the thickness of the enamel layer (*y*‐axis) separated by row tilt (mesial tilt, lateral tilt). These graphs, based on data from 24 incisors, bear a striking similarity to the results obtained from one incisor (Fig. 2C,D), suggesting that the detected rod profile angle changes occur in a highly repetitive manner in mouse incisor enamel (data from one tooth is representative of the pattern present in 24 teeth). In this figure the trends across enamel thickness (*y*‐axis) and regional location (*x*‐axis) are merely smoother and more uniform than in Fig. 2. Circular plots of rod profile angles in the inner enamel layer partitioned by region and by tilt (B). Circular plots of rod profile angles in the inner enamel layer partitioned by region (Fig. 2A) and by tilt (mesial, lateral) for all mandibular mouse incisors. Measurements are in a counterclockwise direction from the 3 o'clock position (0°) with the lateral CEJ situated on the right side and mesial CEJ on the left side of each circle. The four regions of the inner enamel layer are represented by color (1, red; 2, blue; 3, green; 4, violet); points = counts, bars = relative number of observations per color, point with line = mean circular direction (also indicated by *N* ± circular SD). The mean profile tilt angle of rod profiles having a mesial tilt is on average roughly twice as large as rod profiles having a lateral tilt. The regional means and the increase in profile angle from lateral to mesial sides of the inner enamel layer also show this 2 : 1 difference for rod profiles having a mesial tilt compared to those having a lateral tilt. (*N *= total number of rod profiles analyzed in estimating grand means).

The most prominent feature of rodent incisor enamel in cross‐section is the repetitive alternating angulations to rod profiles across the thickness and breadth of the inner enamel layer (Fig. 1BandC, black and red ovals). In any given incisor, the angle at which rod profiles appeared tilted toward the mesial side or the lateral side seemed similar at any given location vertically across the thickness of the inner enamel layer, but there was a noticeable increase in angulation of all rod profiles progressing from the lateral side to the mesial side of the enamel layer (Figs 1 and 2). Rod profiles having a mesial tilt showed the largest linear increase compared with rod profiles having a lateral tilt, which showed only modest increases from lateral to mesial side (Figs 2 and 3). This resulted in the impression that rod profiles with a lateral tilt in section were more horizontal in the lateral region (region 1) and more vertical in the mesial region (region 4), whereas rod profiles with a mesial tilt were more vertical in the lateral region and near horizontal at a much higher angle in the mesial region (essentially inverted mirror images of each other; Figs 2 and 4A). Also, on any given incisor and any region of the enamel layer, the tilt angle showed considerable local variation within the same row, especially in the case of rows having a lateral tilt (Fig. 3). Most of these variations were smoothed out when computed over all 24 incisors analyzed in this study (Fig. 2C,D for one incisor compared with Fig. 4A for all incisors).

In global terms, the angulation grand mean for rod profiles having a mesial tilt in 24 incisors was 104.0° ± 34.2°, whereas for rod profiles having a lateral tilt this was 48.6° ± 25.2° (Fig. 4B). Rod profiles having a mesial tilt showed a mean angle of 58.4° ± 12.5° in the lateral region (region 1) and a mean angle of 147.0° ± 14.1° in the mesial region (region 4) of the enamel layer (Fig. 4B), whereas rod profiles having a lateral tilt show a mean angle of 29.9° ± 52.8° in the lateral region and a mean angle of 75.6° ± 13.5° in the mesial region (Fig. 4B). This resulted in a progressive lateral‐to‐mesial regional change in angulation of about 30° per region for a total of near 90° overall for rod profiles having a mesial tilt but only a 15° step change per region and a 45° change overall, or one‐half, for rod profiles having a lateral tilt (Fig. 4B).

**Figure 5:**
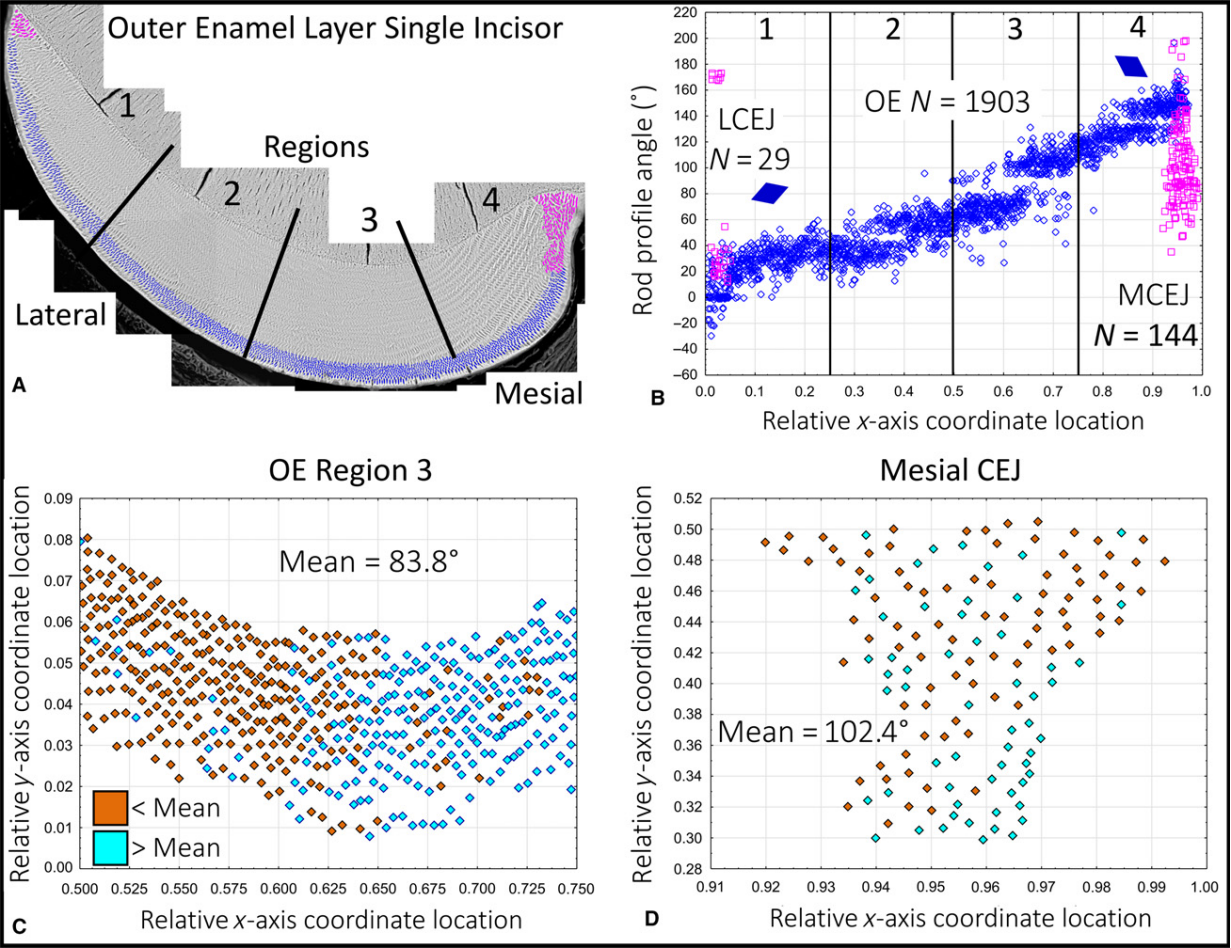
Example data for rod profile angles in the outer enamel layer measured in a single mandibular mouse incisor. Angle data from a single mandibular mouse incisor for rod profiles forming the outer enamel layer (blue) and those located near the mesial and lateral CEJ (magenta). (A) Color map for rod profiles. The enamel layer is partitioned into four equally spaced regions (the same as in Fig. 2). (B) Graph of rod profile tilt angle (*y*‐axis) vs. location across the face of the cross‐section expressed as virtual coordinates (*x*‐axis). The number of rod profiles plotted from (A) are indicated (*N*), as is a schematic representation of the mean rod profile tilt in the outer enamel layer by the diamonds plotted for the lateral (1) and mesial (4) regions. Rod profiles forming the outer enamel layer (blue) show a linear increase in angulations from lateral to mesial sides, while rod profiles situated near the CEJ are more randomly arranged. (C) Graph showing the distribution of rod profile angles in the outer enamel layer of the central labial region (A, region 3, blue) relative to the mean circular angle (less than, brown; greater than, cyan). There is a general trend for rod profile angles to increase in a mesial direction (with some irregularities) but no evidence for a similar change relative to enamel thickness. (D) Graph showing the distribution of rod profile angles near the mesial CEJ (A, region 4, magenta) relative to the mean circular angle (less than, brown; greater than, cyan). Enamel rod profiles at this site appear randomly tilted irrespective of location.

**Figure 6:**
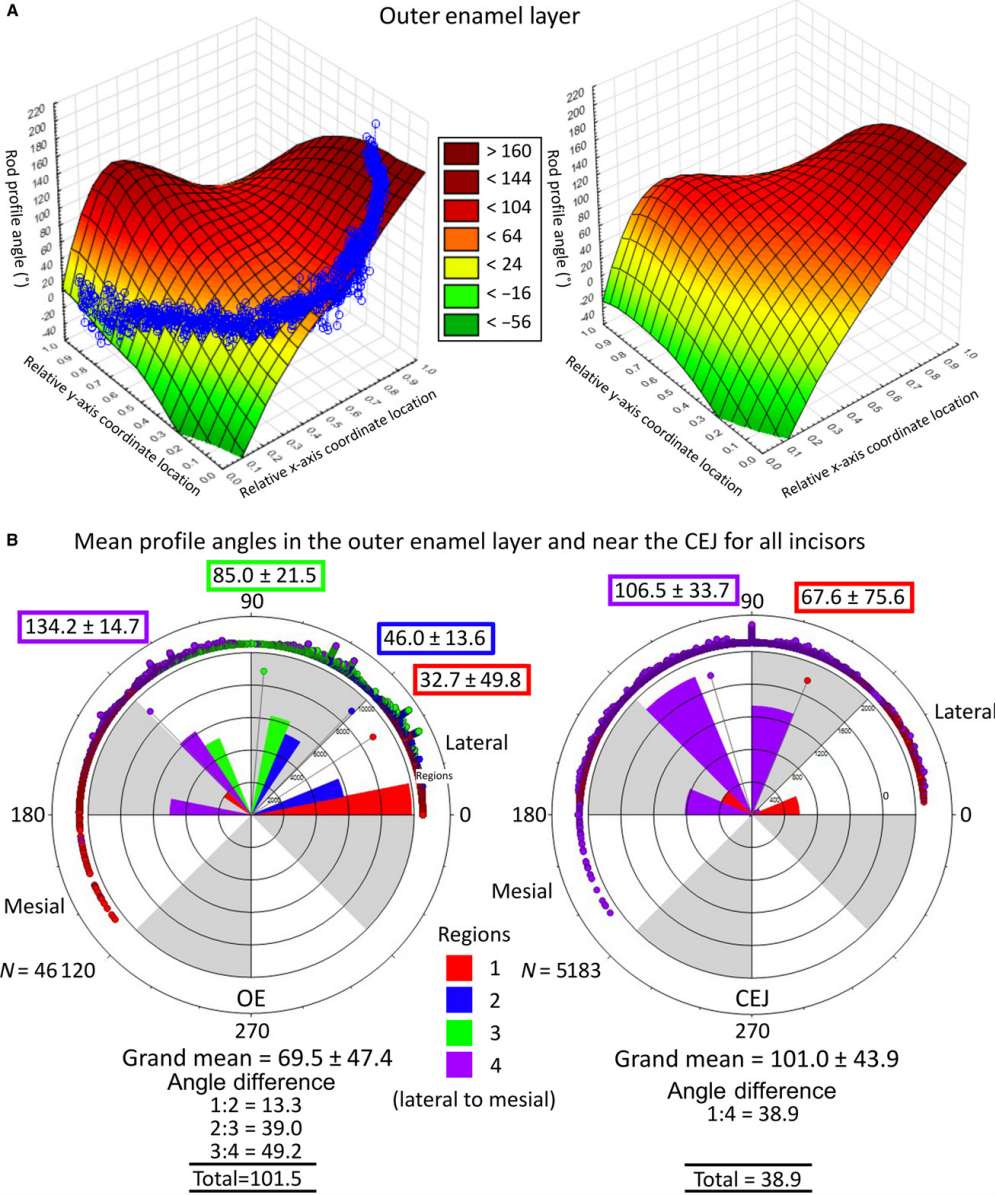
The 3D surface and circular plots of rod profile angles across the width and thickness of the outer enamel layer. The 3D surface plots for a single incisor compared to pooled data from all incisors (A). Distance weighted least‐squared 3D surface plots of rod profile angles across the entire outer enamel layer for one mandibular mouse incisor and for data pooled from all mandibular mouse incisors examined in this study (*z*‐axis) plotted relative to regional location (*x*‐axis) and location within the thickness of the enamel layer (*y*‐axis). These graphs (based on one incisor and 24 incisors) bear a striking similarity to each other, suggesting that the detected rod profile angle changes occur in a highly repetitive manner in mouse incisor enamel (data from one tooth is representative of the pattern present in 24 teeth). Circular plots of rod profile angles across the width and thickness of the outer enamel layer partitioned by region and for rod profiles located near the mesial and lateral CEJ (B). Circular plots of rod profile angles in the outer enamel layer and near the mesial and lateral CEJ partitioned by region (Fig. 5A) for all mandibular mouse incisors. Measurements are in a counterclockwise direction from the 3 o'clock position (0°) with the lateral CEJ situated on the right side and mesial CEJ on the left side of each circle. The four regions of the inner enamel layer are represented by color (1, red; 2, blue; 3, green; 4, violet); points = counts, bars = relative number of observations per color, point with line = mean circular direction (also indicated by numbers ± circular SD). Rod profile angles within the outer enamel layer increase fourfold from lateral to mesial sides of the enamel layer, much greater than is seen for rods forming the inner enamel layer (Fig. 4B) or positioned near the CEJ (right panel). (*N *= total number of rod profiles analyzed in estimating grand means).

Similar regional changes in rod profile angulation from lateral to mesial sides of the enamel layer were also detected for the diamond‐shaped rod profiles forming the outer enamel layer and the irregularly elongated and tilted rod profiles found near the lateral and mesial CEJ (Figs 5 and 6). The changes in rod profile angulation from lateral to mesial regions of the outer enamel layer and features of row angulation distributions across a given region or relative to enamel thickness, resembled changes observed for rod profiles having a mesial tilt within the inner enamel layer, with the exception that the angle changes per region were progressively larger per regional step and the total change in angle from lateral to mesial was greater in the outer enamel layer than in the inner enamel layer (Figs 5B and 6A compared with Figs 2B,C and 4A; Fig. 5C compared with Fig. 3B top right; Fig. 6B compared with Fig. 4B). In global terms, the angulation grand mean for rod profiles forming the outer enamel layer in 24 mandibular mouse incisors was 69.5° ± 47.4° and for rod profiles positioned near the mesial and lateral CEJ 101.0° ± 43.9°. In both cases, deviations in angulation were very high in the lateral region, especially for rod profiles near the CEJ, which showed considerable variation in angles relative to enamel thickness (Figs 5D and 6B).

### Rod angulations relative to the DEJ

**Figure 7:**
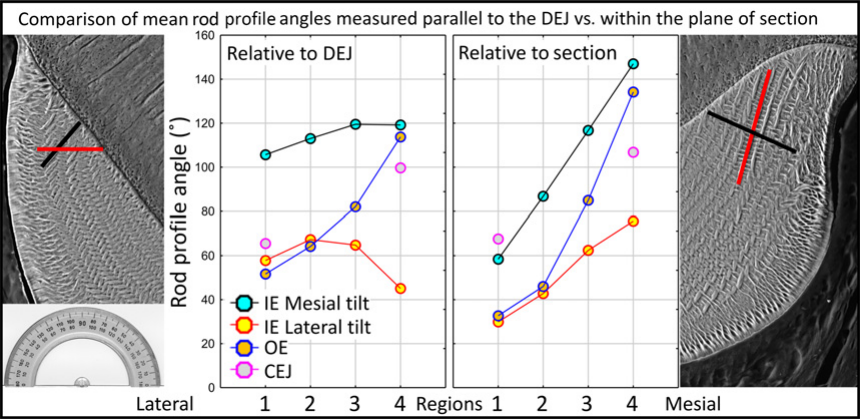
Graphs summarizing rod profile angle relationships across the four regions of the enamel layer as measured relative to the plane of section or to the DEJ. Graphs summarizing rod profile angle relationships across the four regions of the enamel layer (1, lateral; 2, mid lateral, 3, central labial; 4, mesial) within the inner enamel layer (IE), the outer enamel layer (OE) and near the CEJ as measured either within the plane of section (transverse) or relative to cropping box positioned parallel to the DEJ. At the left and right sides of the graphs are BEI images of the lateral and mesial regions (1 and 4) with example rod profiles having a mesial (black) or lateral (red) tilt for orientation purposes. At the left side an image of a protractor is included for reference. Data are from the right and left mandibular incisors of six mice (12 incisors total). Number of rods analyzed, *n* = 50 000 relative to the DEJ and *n* = 92 500 relative to plane of section. Angulation differences from lateral to mesial sides of a cross‐section are much less pronounced for rod profiles forming the inner enamel layer when imaging fields are aligned parallel to the DEJ prior to measurement. Angulation differences are relatively unchanged for rod profiles forming the outer enamel layer and those located near the CEJs irrespective of alignment method.

A somewhat different impression of enamel rod profile angulations and their changes from lateral to mesial sides of the enamel layer was obtained when the imaging plane was aligned parallel to the DEJ prior to making the angle measurements (Figs 1C and 7). One difference was that rod profile angulations at the lateral side of the enamel layer appeared larger, whereas those at the mesial side appeared reduced compared with measurements made relative to the plane of section (Fig. 7). This resulted in greatly reduced changes in absolute angles progressively from lateral to mesial sides across the enamel layer. Secondly, rod profiles within the inner enamel layer having a mesial tilt showed only a modest increase in angulation from lateral to mesial sides, whereas rod profiles having a lateral tilt showed little change but there was a decrease in rod profile angulation approaching the mesial side of the inner enamel layer (Fig. 7). Rod profiles forming the outer enamel layer and those located near the CEJ show similar trends for increasing angulations progressing from lateral to mesial sides of the enamel layer, but only about one‐half the absolute change in angulation compared with measurements made relative to plane of section (Fig. 7).

### Angulation changes between alternating rows

**Figure 8:**
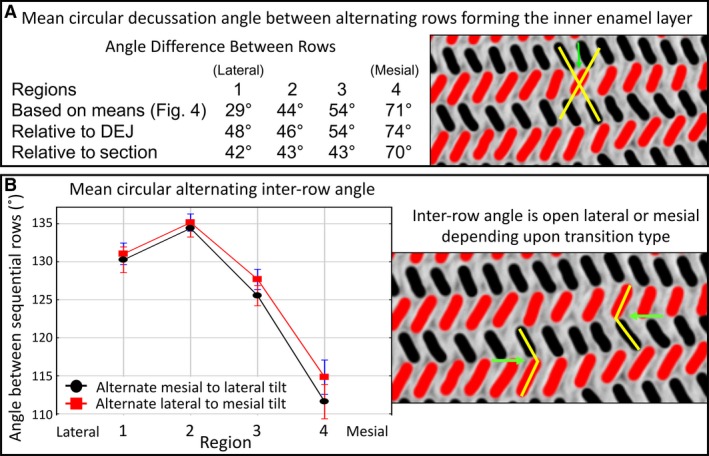
Decussation and alternating inter‐row angles. Decussation angle between rows alternating between mesial and lateral tilts computed from mean angles or as measured relative to the plane of section or to the DEJ (A). Decussation angle between rows with alternating mesial and lateral tilts measured across the four regions of the inner enamel layer (1, lateral; 2, mid lateral, 3, central labial; 4, mesial). At the left side of the table is a small cropped area of the inner enamel layer from a color map (black = mesial tilt; red = lateral tilt) illustrating with the yellow lines the decussation angle (green arrow). Number of alternating rows analyzed, *n* = 2974 from 24 mandibular incisors. These results suggest that measurements of the decussation angle are independent of plane of section and tooth curvature (i.e. this variable is row‐dependent rather than dependent on the plane of section or alignment). Considering the manner in which row development occurs in the incisor (see Fig. 10), these data further suggest that the decussation angle between rows with alternating tilts increases sharply as the wave of differentiation spreads mesially from the central labial side of the tooth (region 3 to 4), but the decussation angle changes little as the wave spreads laterally (from region 3 to 2, then to region 1). (B) Alternating inter‐row angle measured across the four regions of the inner enamel layer (1, lateral; 2, mid lateral, 3, central labial; 4, mesial). At the left side of the table is a small cropped area of the inner enamel layer from a color map (black = mesial tilt; red = lateral tilt) illustrating with the yellow lines the inter‐row angle as the alternation from mesial (black) to lateral (red) tilt or from lateral (red) to mesial (black) tilt (green arrows). Number of alternating rows analyzed, *n* = 480 per tilt category from 12 mandibular incisors. There are no clear differences between the red points and black points within the same region. As expected, the inter‐row angle is smallest in the mesial region (4) where the decussation angle between alternating rows is the greatest (top panel).

The alternating mesial and lateral tilts of rod profiles within the inner enamel layer creates two angles that can be quantified. One is the simple angle difference between sequential rows, or the decussation angle (Moinichen et al. 1996; Fig. 8A). This angle can be computed from grand means (Fig. 4B) or from actual measurements of angle differences on a row‐by‐row basis (e.g. Figs 3 and 7). Results from these angle measurements are surprisingly consistent, except for an underestimation of angle difference in the lateral region of the inner enamel layer based upon grand means (Fig. 8A, region 1). The trend is for a gradual increase in decussation angle between alternating rows until the mesial side of the inner enamel layer, where the angle difference increases sharply (Fig. 8A). The second angle created when rows of opposite tilts cross one another, is the wide angle created where the two rows abut, herein termed the alternating inter‐row angle (Fig. 8B). This angle can be measured either as a transition from rows having a mesial tilt to rows having a lateral tilt (Fig. 8B, black‐to‐red with angle open to lateral side) or between rows having a lateral tilt to rows having a mesial tilt (Fig. 8B, red‐to‐black with angle open to mesial side). The alternating inter‐row angle was consistently slightly higher for lateral‐to‐mesial row tilt transitions (not significant), but in both cases the angle decreased from a maximum near 135° in region 2 (mid lateral) to a minimum near 110° in region 4 (mesial) in progressing from the lateral to mesial sides of the inner enamel layer (Fig. 8B). These results suggest that measurements of angle differences between rows are fairly independent of plane of section and tooth curvature (i.e. this parameter is row‐dependent rather than dependent on plane of section or alignment).

### Spacing of rods within the enamel layer

**Figure 9:**
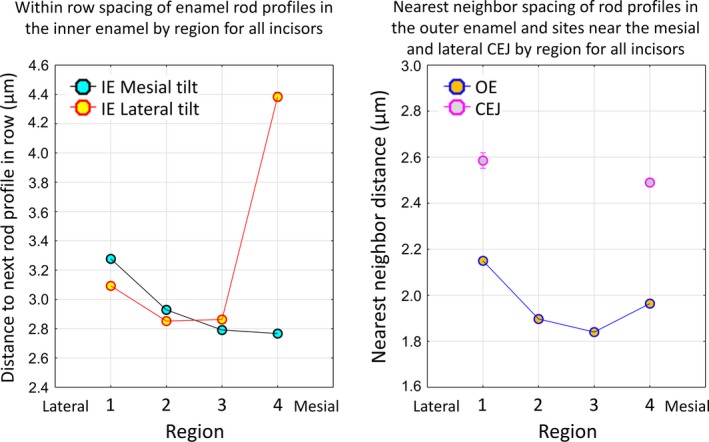
Graphs summarizing distance between rod profiles (spacing) across the four regions of the enamel layer within the inner and outer enamel layers and near the CEJ. Graphs summarizing distance between rod profile (spacing) across the four regions of the enamel layer (1, lateral; 2, mid lateral, 3, central labial; 4, mesial) relative to the inner enamel layer (IE), the outer enamel layer (OE) and near the CEJ. Data are from 24 mandibular incisors of 18 mice Inner enamel: *n* = 62 986 mesial tilt, *n* = 56 337 lateral tilt; outer enamel: *n* = 46 120; CEJ: *n* = 5183. The tightest packing together of rod profiles occurs in the central labial region (3) of the outer enamel layer (right panel) followed by the central labial and mesial (4) regions of the inner enamel layer for rod profiles having a mesial tilt (left panel). The widest spacing of rod profiles occurs in the mesial region of the inner enamel layer for rod profiles having a lateral tilt (left panel). Rod profile spacing in both the inner and outer enamel layers shows a trend to increase in a lateral direction (region 3 toward region 1) and to increase in a mesial direction, but only within the outer enamel layer (region 3 toward region 4). Rod profile spacing near the CEJ is similar at the mesial and lateral sides and is intermediate in distance between spacing seen in the inner and outer enamel layers.

**Figure 10:**
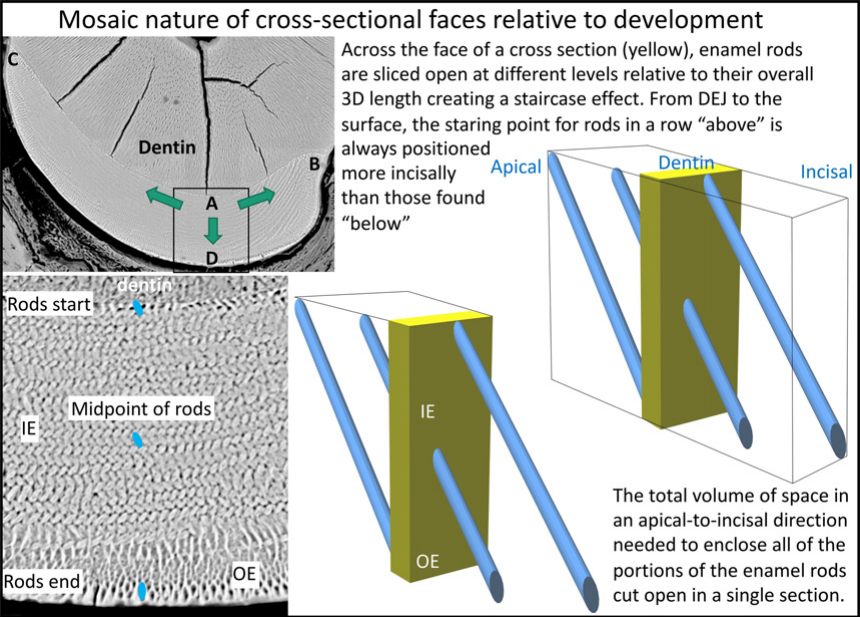
Schematic illustration of the multidirectional developmental pattern for enamel and the step‐like arrangement of enamel rods created relative to the cross‐sectional plane of the mandibular mouse incisor. Low‐magnification BEI image of a cross‐section of the enamel layer covering the labial side of a mandibular mouse incisor (top left side). The boxed area is shown at higher magnification at the bottom. On the right side are schematic drawings illustrating how enamel rods project into or out of the plane of section to varying degrees depending upon their location across the thickness of the enamel layer. Enamel rod profiles, each representing a single 2D slice from the much larger 3D enamel rod, found near the DEJ are the most apical starting points for rods projecting mostly in an incisal direction from the plane of section, whereas rod profiles seen near the outer surface are cut at the incisal end of rods projecting in an apical direction backwards into the tissue block. Rod profiles seen in the middle of the enamel layer would have half their 3D length projecting apically and the other half projecting incisally, with all other profiles inbetween projecting predominately incisally (above midpoint) or apically (below midpoint). During development, enamel formation begins near the DEJ at the central side of the tooth (boxed area, location A) and spreads as a wave in a mesial direction (arrow to location B) and lateral direction (arrow to location C) as the enamel layer increases in thickness by appositional growth (arrow to location D) (Smith & Warshawsky, 1976). A cross‐section of the incisor therefore represents a composite image built up over time of the creation of lamellar sheets of rods stacked in a step‐like arrangement one behind the other with alternating tilts. This formative process spreads as a wave to the mesial and lateral sides of the labial surfaces so that location A begins its development before location B, followed by location C, thereby creating a time composite of development across the whole enamel layer. The thin ring of rod profiles abutting the DEJ and those abutting the outer surface define the entire volume of 3D enamel rod space sampled by the cross‐section.

The last feature of gross 2D arrangement of enamel rods examined in cross‐sections was the distance between adjacent rod profiles forming rows within the inner enamel layer and, since they are not arranged in clearly definable rows, the nearest neighbor distances between rod profiles forming the outer enamel layer and those positioned near the CEJ (Fig. 9). The side‐by‐side spacing of enamel rod profiles across rows having a mesial tilt showed a gradual compression, progressing from lateral to mesial sides of the inner enamel layer (Fig. 9). A similar trend for shortening of the between‐rod profile spacing was seen in rows having a lateral tilt, but this occurred only between region 1 (lateral) and region 2 (mid lateral). There was no change in the between‐rod profile spacing from region 2 (mid lateral) to region 3 (central labial), but the spacing distance between rod profiles increased by almost twofold in moving from region 3 (central labial) to region 4 (mesial) of the inner enamel layer (Fig. 9). Rod profiles forming the outer enamel layer were more tightly packed together compared with rod profiles present within the inner enamel layer, which thereby allowed ~ 30% of all enamel profiles (Table 1) to be packed into 20% of the cross‐sectional area of the enamel layer (Fig. 1, Table 1). The outer enamel rod profiles showed the same trend as rod profiles in the inner enamel layer to become more closely spaced between the lateral and central regions of the outer enamel layer (regions 1–3), followed by an increase in spacing in the mesial region (Fig. 9). Rod profiles located near the lateral and mesial CEJ showed only slight differences in their nearest neighbor distances (Fig. 10). They were more tightly packed together compared with rod profiles forming the inner enamel layer but were more dispersed compared with rod profiles forming the outer enamel layer (compare Figs 10 and 9). We found no evidence for any major change in packing density of the enamel rods relative to enamel thickness, only a significant difference relative to regional division of the enamel layer.

## Discussion

It was initially anticipated that attempts to count every enamel rod profile present in cross‐sections cut from mouse mandibular incisors would prove very difficult because of uncertainties at many sites in distinguishing clearly the boundaries of rod profiles from their surrounding inter‐rod material. The most problematic sites include where the innermost ends of the enamel rods are located in close proximity to the DEJ, the boundary region where the inner enamel portions of rods transition into smaller and more diamond‐shaped outer enamel portions (Warshawsky & Smith, 1971), and where the outer enamel portions of the rods terminate near the enamel surface (Fig. 1B). Enamel rods also appear disorganized and their outlines obscure in areas approaching the CEJ at the mesial and lateral sides of the enamel layer (Moinichen et al. 1996). As it turned out, defining boundaries of rod profiles and counting them was not a major obstacle (Table 1). What proved the most problematic was the technical issue of obtaining high clarity etchings of polished tooth slices. This proved difficult to control, and often repeat polishing and etching of tooth slices was required to obtain the best imaging of the exposed enamel rod profiles. Both standard mode and backscattered mode SEM were acceptable for creating high‐magnification montage maps of the enamel layer, but inter‐rod material was more prominent, and caused more interpretive interference in the former and this was the reason why the latter approach was chosen as the method of choice for this investigation.

All past reports of enamel rod distributions in cross‐sections of rodent incisor enamel have been qualitative in nature and have focused on features such as enamel thickness, general organization of rows of rods within the inner enamel layer or various angles at which the decussating enamel rods cross one another or are tilted relative to the plane of eruption, the outer enamel layer, and/or the enamel surface (Warshawsky, 1971; Jodaikin et al. 1984; Moinichen et al. 1996). Moinichen et al. (1996) published one of the most detailed and informative investigations about the 2D organization of the enamel layer in mouse incisor. These workers did not attempt to quantify the number of enamel rod profiles cut open in cross‐sections either globally or on a regional basis, but they noted several important details about the structural arrangement of enamel rods in mouse incisor enamel pertinent to findings in this study.

### Enamel thickness

Moinichen et al. (1996) reported that enamel thickness in the central labial region was 95 μm on the erupted portion of mandibular incisors from 5‐week‐old Balb/c albino mice. We found enamel thickness was 121 ± 2.7 μm in preeruptive enamel near the gingival margin in 7‐week‐old C57BL/6 pigmented mice (Table 1). There are several possible reasons for this 20% discrepancy in thickness measurements, including strain differences (Li et al. 2013), but the more likely explanation is related to age and site of sampling for imaging. In preeruptive and early posteruptive rodent incisors, the diameter of the developing tooth at its incisal end is always smaller than the part of the tooth buried more apically (Sehic et al. 2009). This is due to growth changes in diameter and length of the incisors which take time to stabilize. We found in preliminary studies that these growth changes in size take around 7 weeks to terminate. This is confirmed by a later report from the same research group noted above that enamel thickness in adult wild type mice (> 2 months in age) was 128 ± 8 μm (Risnes et al. 2005), almost identical to the thickness value we obtained.

### Incisal tilt angle of enamel rods

The enamel rods in rodent incisors do not travel in straight lines from the DEJ to the surface but are all angled in a forward (incisal) direction, which is why they appear within the inner enamel layer in profile as elongated ovals in cross‐sections. The incisal tilt angle is not fixed to one specific value but varies by species (e.g. rat vs. mouse), tooth type (maxillary vs. mandibular incisor), and location within the enamel layer (outer enamel layer vs. inner enamel layer). Moinichen et al. (1996) reported that in mandibular mouse incisors the alternating rows of rods forming the inner enamel layer are tilted incisally by 45° relative to the DEJ, whereas the outer enamel portions of the rods are angled more broadly at 88°, thereby creating an angle of 12° to the enamel surface. In this study, we used sections cut only in the cross‐sectional plane and therefore could not estimate the incisal tilt angle.

### Rod decussation angle

There has been considerable disagreement in the literature regarding the angle at which the alternating rows of rods cross one another traveling in either a mesial or lateral direction across the thickness of the inner enamel layer in rat and mouse incisors, from as little as 30° (Moinichen et al. 1996) to as much as 90° (Warshawsky, 1971). As with rod incisal tilt angle, differences by species and tooth type have been noted for decussation angles in mandibular mouse incisors (Von Koenigswald, 1985; Moinichen et al. 1996; Martin, 1999; Vieytes et al. 2007), but by far the greatest differences were reported by Moinichen et al. (1996) relative to positional location of rows across the thickness of the inner enamel layer, that is, a decussation angle in mandibular mouse incisors of 30° near the DEJ, an angle of 60° in the middle portion of the inner enamel layer, and a decussation angle of 80° near the boundary of inner and outer enamel layers. The average decussation angle observed in this study based on grand means for row tilts (Figs 4B and 8A) was comparable to a grand mean that can be computed from the range of decussation angles reported by Moinichen et al. (1996): 104° − 49° = 55° vs. circular mean of 30° + 60° + 80° = 57°. We were unable, however, to find a trend for a 30° to 80° decussation angle increase across the thickness of the inner enamel layer relative to either single incisors (Fig. 2C,D, *y*‐axis of graphs) or in data pooled from multiple incisors (Fig. 5, *y*‐axis of paired graphs). Instead we found that the greatest change in rod tilt angulations and computed decussation angle relative to the two opposing rod tilts within the 2D plane of a cross‐section, occurred in the *x*‐axis direction (lateral‐to‐mesial side of the enamel layer) rather than in the *y*‐axis direction (thickness), especially relative to rows having a mesial tilt (Figs 2, 3, 4 and 7). Of interest in this study was the finding that increases in rod profile angulations from the lateral to mesial side of the enamel layer (the direction rows of rods are organized into), and to a lesser extent from DEJ to the surface, did not occur in a smooth, regular fashion but instead the transitions were often noisy, with increases and decreases in tilt angles intermixed at certain local sites even within the same row (e.g. Fig. 3). Increases in rod angulations were apparent only by computing grand means with a broader partition of the enamel layer (e.g. divisions by region; Figs 2, 3, 4). Of interest was the additional finding that the increase in rod angulation from lateral to mesial side of the enamel layer also applied to the diamond‐shaped outer enamel portions of the enamel rods (Figs 5, 6, 7). In global terms, the change in angulation of tilt of the outer enamel portion of the enamel rods resembled changes detected for rod profiles having a mesial tilt within the inner enamel layer, but in regional terms the grand means computed for rod angulation in the lateral one‐half of the enamel layer (regions 1 and 2) resembled grand means computed for rod profiles having a lateral tilt, whereas the means resembled rod profiles with a mesial tilt in the mesial one‐half of the enamel layer (regions 3 and 4; Figs 4, 5, 6, 7). To the knowledge of the authors, these characteristics of rod profile angulation changes within the outer enamel layer have not previously been reported, including by Moinichen et al. (1996), who focused on differences in the incisal tilt angle for the outer enamel portions of rods, which we did not investigate in this study.

### Arrangement of enamel rods near the CEJ

Moinichen et al. (1996) are among few investigators that have drawn attention to the unusual appearance and arrangements of enamel rods located at sites near the lateral and mesial CEJ in rat and mouse incisors. Here, row arrangements are obscure, rod tilts are disorderly, organization of rods into inner and outer enamel portions is difficult to define, and inter‐rod type enamel appears more prominent (Figs 1, 5, and 7). Of interest in this study were the findings that the mean tilt angle of these disorganized rods followed the same regional trend to be greater at the mesial side than at the lateral side of the enamel layer, and that almost five times more rod profiles per cross‐section were detected mesially than laterally (Fig. 7, *n‐*values), presumably in part reflecting the fact that the whole enamel layer is thinnest near the lateral CEJ (Fig. 1). The CEJs are the last sites to develop during the secretory stage, and the disorganization of rods formed in these areas may reflect a barrier function that occurs by ameloblasts to allow smoothing at the outermost edges of the enamel layer that would otherwise show a step‐like ragged arrangement relative to terminations of rows having alternating tilts.

### Rod spacing

The packing density of enamel rods within the enamel layer has not been investigated to any extent by past investigators. Figure 9 shows clearly that, like rod tilt angles, there is regional variation in the way enamel rods are packed together, but with opposite trends. That is, within the inner and outer enamel layers, the rods are spaced farther apart in the extreme lateral region (region 1) than in the central labial region (region 3). For rod profiles within the inner enamel layer having a mesial tilt there is further shortening of the distance between neighbors from the central labial region to the mesial region (region 4) of the enamel layer, whereas for rod profiles having a lateral tilt and rod profiles forming the outer enamel layer, neighboring rods are spread farther apart from one another (Fig. 9). Comparing Fig. 7 with Fig. 9,there appears on a regional basis to be an inverse correlation between rod tilt angle relative to the DEJ and rod spacing at all sites within the enamel layer. If the angle increases from one region to the next, the rod spacing decreases, if the angle stays unchanged, so does the rod spacing, and if the rod angle decreases, the rod spacing increases (Figs 7 and 9). This could reflect structural concessions in that the enamel is highly curved from the lateral to mesial side along the DEJ and varies in thickness from the lateral to mesial side. The highest density of rods is concentrated in the central labial region (region 3), possibly representing a structural feature that allows the incisor tip to maintain a shovel‐shape, with the sharp tip positioned in the central labial plane (Von Koenigswald, 1985; Kuang‐Hsien Hu et al. 2014).

### Interpreting enamel rod arrangements in cross‐sections

The global arrangement of enamel rods within the enamel layer of rodent incisors is clearly complex and has proven difficult to conceptualize in histological sections. One aspect of the global arrangement of enamel rods that past investigators have not fully taken into accounted is the mosaic nature of rod arrangements in terms of time and space. That is, while there is a high degree of symmetry and continuity in the way in which enamel rods fill the enamel layer, the packing of these enamel rods as seen in a typical cross‐section of mature rodent incisor enamel presents a composite image built up over time to create its final amalgamated form in three‐dimensional space (Fig. 10; Smith & Warshawsky, 1976). In terms of space, rod profiles seen near the DEJ represent the starting point of rods projecting forward almost in their entirety out of the plane of section, rod profiles situated the middle of the enamel layer represent rods cut at their midpoints with half the rod projecting backwards into block of tissue and the other half projecting forwards out of the plane of section, and rod profiles seen near the surface in the cross‐section represent the endpoints of rods projecting almost in their entirety backwards into the block (Fig. 10). Hence, the thickness of the enamel layer, which develops over the duration of the secretory stage by an appositional growth process, requires the coordinated efforts of all those ameloblasts needed to maintain continuity across the boundaries delineated between the lateral and mesial CEJ (Smith & Warshawsky, 1976). This is one of the main reasons for wanting to know how many rod profiles are present within a representative cross‐section of rodent incisor enamel, as it puts into perspective the basic unit (cohort) of ameloblasts needed to renew the enamel layer over time (Smith & Warshawsky, 1977). This investigation has suggested that this involves the participation of approximately 7200 ameloblasts per renewal cycle (Table 1).

There are two additional implications of this step‐like development of enamel rods relative to the longitudinal axis of the incisor. The first is that ameloblasts located at the enamel surface at the end of the secretory stage have no formative relation to 99.9% of the enamel rods situated vertically between them and the DEJ (as would be seen a cross‐section of the incisor). The enamel rods they have produced all project BACKWARDS (apically) from the site where they are currently located (Fig. 10). The second implication is that throughout the maturation stage a given group of ameloblasts is assisting the final mineralization in the vertical plane of portions of thousands of enamel rods they did not secrete.

There is a second developmental axis in rodent incisor enamel formation besides the one associated with appositional growth (progressive development of enamel thickness from DEJ to surface). This also requires time and space to develop fully and involves the spread of a wave of differentiation from the point of maximum convexity along the central labial aspect of the tooth (equivalent to the incisal edge on a human incisor) in mesial and lateral directions until a termination point is reached at what becomes the mesial and lateral CEJ (equivalent to the cervical area on a human incisor; Fig. 10; Smith & Warshawsky, 1976). It takes more time for the wave of differentiation to terminate laterally than mesially, thereby creating the side‐to‐side asymmetry typical of rodent incisor enamel (Moinichen et al. 1996). It is prior to or during the movement of this wave that the row arrangements with alternating rod tilts characteristic of the lamellar sheet arrangement of rods forming the inner enamel layer are established (Smith & Warshawsky, 1976; Cox, 2013). It is presumably the time delay between the start of formation of enamel in the central labial region and the completion of the wave of ameloblast induction at the sites, which become the mesial and lateral CEJs, that ultimately creates the step‐like pattern of rod arrangement in the enamel layer.

## Conflict of interest

The authors declare no conflict of interest.

## Author contributions

This study was designed principally by C.E.S. with contributions by J.P.S. and J.C‐C.H. The incisors were prepared, sectioned, polished, and BEI‐imaged by Y.H. J.C‐C.H. oversaw the growth and mating of mice. Data analysis and creating the first draft of the manuscript and figures was performed by C.E.S. The figures were modified by J.P.S. The manuscript was critically reviewed by J.C‐C.H. and J.P.S.
